# Plasma Biomarkers and Immune Checkpoint Inhibitors in Non-Small Cell Lung Cancer: New Tools for Better Patient Selection?

**DOI:** 10.3390/cancers11091269

**Published:** 2019-08-29

**Authors:** Adrien Costantini, Paul Takam Kamga, Coraline Dumenil, Thierry Chinet, Jean-François Emile, Etienne Giroux Leprieur

**Affiliations:** 1 Department of Respiratory Diseases and Thoracic Oncology, APHP—Hôpital Ambroise Paré, 92100 Boulogne-Billancourt, France; 2 EA 4340 BECCOH, UVSQ, Université Paris Saclay, 92100 Boulogne-Billancourt, France; 3 Department of Pathology, APHP—Hôpital Ambroise Pare, 92100 Boulogne-Billancourt, France

**Keywords:** non-small cell lung cancer, plasma, biomarkers, immune checkpoint inhibitor

## Abstract

Immune checkpoint inhibitors (ICIs) have transformed the treatment landscape for patients with non-small cell lung cancer (NSCLC). Although some patients can experience important response rates and improved survival, many others do not benefit from ICIs developing hyper-progressive disease or immune-related adverse events. This underlines the need to select biomarkers for ICIs use in order to better select patients. There is currently no universally validated robust biomarker for daily use of ICIs. Programmed death-ligand 1 (PD-L1) or tumor mutational burden (TMB) are sometimes used but still have several limitations. Plasma biomarkers are a promising approach in ICI treatment. This review will describe the development of novel plasma biomarkers such as soluble proteins, circulating tumor DNA (ctDNA), blood TMB, and blood microbiome in NSCLC patients treated with ICIs and their potential use in predicting response and toxicity.

## 1. Introduction

Immune checkpoint inhibitors (ICIs) are humanized monoclonal antibodies that mainly target programmed death 1 (PD-1), programmed death-ligand 1 (PD-L1), or cytotoxic T-lymphocyte-associated protein 4 (CTLA-4). They are currently transforming treatment strategies across numerous cancer types and especially advanced non-small cell lung cancer (NSCLC) [1,2,3,4,5,6,7].

PD-L1 and programmed death-ligand 2 (PD-L2) are membranous proteins expressed by malignant cells that interact with PD-1 expressed by T-cells. When PD-L1/PD-L2 and PD-1 bind, the T-cells’ cytotoxic anti-tumor activity is down-regulated. By blocking the interaction between PD-L1 and PD-1, anti-PD-1 and anti-PD-L1 antibodies restore cytotoxic immune response.

Although this approach has changed the treatment strategy in advanced NSCLC (first-line setting, second-line setting, and beyond) as well as in locally advanced NSCLC (consolidation setting after chemo-radiotherapy) [1,2,3,4,5,6,7,8], a large proportion of patients will not benefit from ICIs and patient selection is still a challenge. At the moment, immunohistochemistry (IHC) is the standard biomarker used to evaluate PD-L1 expression on tumor cells and not only informs treatment decisions but also regulatory approval, with a threshold at 50% of positive tumor cells for pembrolizumab in first-line treatment for advanced NSCLC, and 1% for pembrolizumab in second-line or durvalumab in consolidation after chemo-radiotherapy in locally advanced NSCLC.

However, the use of PD-L1 as a predictive biomarker remains challenging, as some patients experience tumor response with low/negative PD-L1 IHC expression [1,2,4,8]. There is also a spatial intra-tumor and inter-tumor heterogeneity [9,10,11,12,13,14,15] as well as temporal variation of PD-L1 expression, especially after chemotherapy [16,17].

There is currently an unmet need for new biomarkers in order to better select patients who will benefit from ICIs. Research is currently focusing on tissue-based biomarkers such as PD-L1 IHC or the tissue tumor mutational burden (tTMB) but also on plasma biomarkers. The advantages of plasma biomarkers are numerous: plasma is easily accessible, less invasive than tissue biopsies, and represents the entire tumor burden present within the patient.

The aim of this review is to describe the development of novel plasma biomarkers such as soluble proteins, circulating tumor DNA (ctDNA), blood TMB (bTMB), and blood microbiome in NSCLC patients treated with ICIs, and their potential use in predicting response and toxicity.

## 2. Circulating PD-L1

PD-1 is an immunoglobulin superfamily type 1 transmembrane glycoprotein consisting of 288 amino acids. It is expressed on tumor cells and different immune cells such as T cells [18]. PD-L1 is a ligand of PD-1 and can be expressed in two forms: membrane-bound (mPD-L1) and in a soluble form (sPD-L1), which is present in the peripheral blood of patients with solid tumors. It has been shown that patients affected with lung cancer have higher levels of sPD-L1 than healthy controls [19].

### 2.1. Prognostic Role of sPD-L1 in NSCLC

Several studies have described the prognostic role of sPDL-L1 in NSCLC. Zhang et al. included 109 patients with advanced NSCLC and 65 healthy controls. Enzyme-linked immunosorbent assay determination (ELISA) was used to measure sPD-L1 levels [19]. The authors determined a sPD-L1 cut-off at 0.636 ng/mL to distinguish patients with high (n = 61) and low (n = 48) sPD-L1 concentrations. When comparing overall survival (OS) between these two groups, it was found that patients with low sPDL-L1 concentrations had longer median OS: 26.8 months vs. 18.7 months for patients with high sPD-L1 concentrations (*p* < 0.001). Finally, when comparing sPD-L1 levels and clinical-pathological features it was found that sPD-L1 was associated with abdominal organ metastasis (*p* = 0.004). No other statistically significant association was observed.

Another study [20] included 96 patients with advanced or post-surgical recurrent lung cancer. ELISA was used to measure sPD-L1 levels (mean sPD-L1 concentration of 6.95 ng/mL). A cut-off was determined at 7.23 ng/mL to separate patients into high (n = 40) and low (n = 56) sPD-L1 concentrations. Again, patients with high sPD-L1 concentrations had shorter OS than patients with low sPD-L1 concentrations: 13.0 months vs. 20.4 months (*p* = 0.037). No correlation was noted between sPD-L1 levels and clinical-pathological features. However, when multivariate analysis was performed, patient age, performance status (PS), use of steroids, and sPD-L1 levels were independently associated with survival.

These two studies bring relevant data: sPD-L1 is present in the plasma of healthy controls and at higher levels in patients with advanced NSCLC. Higher sPD-L1 concentrations seem to be associated with worse survival. However, these studies were performed before the immunotherapy era and although patient treatment was not specified it is likely that they received chemotherapy. Furthermore, we do not have the detail as to when the plasma sample was drawn (at diagnosis or during chemotherapy) rendering the interpretation of these findings difficult.

Two reviews, both associated with a meta-analysis [21,22], assessed the prognostic significance of sPD-L1 in 1102 and 1040 patients respectively with advanced solid tumors. The two meta-analyses did not include the same studies and different cancer types were present (hepatocellular carcinoma, diffuse large B cell lymphoma, NSCLC, gastric adenocarcinoma, biliary tract cancer, multiple myeloma, renal cell carcinoma). However, both studies showed that patients with high sPD-L1 levels had shorter OS than patients with low sPD-L1 levels: hazard ratio (HR) at 1.60 (95% CI: 1.21–1.99, *p* < 0.01) and 2.26 (95% CI: 1.83–2.80, *p* < 0.001), respectively.

### 2.2. sPD-L1 in NSCLC Patients Treated with Radiotherapy

sPD-L1 has also been evaluated in patients with locally advanced or inoperable NSCLC treated with thoracic radiotherapy (TRT) alone or with concurrent chemo-radiotherapy [23]. Zhao et al. performed dynamic measures of sPD-L1, at diagnosis (before initiating TRT), during TRT (week 2 and week 4), and after radiotherapy treatment (within three months of the last TRT treatment day). The study included 126 patients and found that sPD-L1 levels were significantly lower at week 2 and week 4 when compared to baseline (*p* < 0.001 and *p* < 0.001). As had been previously reported, low sPD-L1 concentrations at diagnosis were associated with longer OS as compared to high sPD-L1 concentrations. In this study, the optimal cut-off for sPD-L1 at diagnosis was 0.0965 ng/mL and the median OS for patients with low sPD-L1 levels was 27.8 months vs. 15.5 months (*p* = 0.005) for other patients.

### 2.3. sPD-L1 in NSCLC Patients Treated with ICIs

Several recent publications have focused on sPD-L1 in patients treated with ICIs. Okuma et al. included 39 Japanese patients with stage IV or recurrent NSCLC treated with an anti-PD-1 antibody (nivolumab, 3 mg/kg every two weeks) in the second-line setting or more [24]. Plasma samples were drawn at baseline and sPD-L1 levels were measured by ELISA. The median sPD-L1 concentration was 2.24 ng/mL and the sPD-L1 cut-off to differentiate low and high sPD-L1 concentrations was 3.357 ng/mL. Patients with high sPD-L1 concentrations had shorter OS and shorter time to treatment failure (TTF) compared to patients with low sPD-L1 concentrations: respectively 7.20 months vs. not reached (*p* = 0.040) for OS and 1.48 months vs. 5.36 months (*p* = 0.032) for TTF. Furthermore, the overall response rate (ORR) with nivolumab was greater in the group with low sPD-L1 concentrations compared to high sPD-L1 concentrations (59% vs. 25%, *p* = 0.0069). In univariate analysis, there was a statistically significant difference in sPD-L1 concentrations between patients achieving CR/PR/SD compared to patients presenting with progressive disease (PD) (*p* = 0.0066), no other difference with regards to clinical-pathological features was found. In multivariate analysis, sPD-L1 levels (low vs. high) remained associated with TTF (HR: 0.37, *p* = 0.041).

We recently reported [25] a single-center study that included 43 patients with advanced NSCLC treated with nivolumab (3 mg/kg every two weeks) in second-line treatment or more. Plasma was not only drawn before nivolumab initiation but also at the first tumor evaluation (two months), giving a dynamic view of sPD-L1 variations during treatment. ELISA was performed on plasma samples drawn at different time points during nivolumab. sPD-L1 concentrations at the first tumor evaluation were significantly higher in non-responders with a median value of 67.64 pg/mL (IQR 46.36–75.14) compared to 32.94 pg/mL (IQR 24.89–58.91) in responders (*p* = 0.031). In the same way, it was found that patients who had a clinical benefit from nivolumab (CR, PR, or stability according to RECIST lasting six months or more after nivolumab initiation) had significantly lower sPD-L1 concentrations at the first tumor evaluation under nivolumab (median value of 0.06764 pg/mL) than patients without clinical benefit (median value of 0.03414 pg/mL, *p* = 0.024). A cut-off level of sPD-L1 concentration at the first tumor evaluation was determined at 0.03397 ng/mL. Patients with high sPD-L1 concentrations at two months of nivolumab had a significantly shorter median PFS (2.2 months vs. 11.8 months, *p* = 0.041) and shorter median OS (6.2 months vs. NR, *p* = 0.087) than other patients. Moreover, patients who had an increase in sPD-L1 concentrations between nivolumab initiation and first tumor evaluation had worse ORR (17% vs. 68%, *p* = 0.005), lower rates of clinical benefit (10% vs. 47%, *p* = 0.047), shorter median PFS (1.8 months vs. 6.5 months, *p* = 0.008), and shorter median OS (5.4 months vs. NR, *p* = 0.028) than patients who had decreasing or stable sPD-L1 concentrations.

These different studies bring consistent data relative to the value of sPD-L1 as a prognostic biomarker, at first in patients treated with CT but now also in patients receiving ICIs. Furthermore, some results suggest that sPD-L1 could be used to monitor the efficacy of ICIs and might help to anticipate which patients will benefit from the treatment. These different studies are summarized in Table 1. A major limitation in the translation to clinics for sPD-L1 is the current absence of standardization of sPD-L1 measurement, with several ELISA kits used in the studies, with different thresholds.

### 2.4. Source and Biological Activity of sPD-L1

The studies presented above have shown that a soluble form of PD-L1 can be detected in the blood/plasma of healthy controls as well as in that of patients affected with NSCLC. However, several questions remain, such as the biological origin and activity of sPD-L1.

Frigola et al. [18] demonstrated that sPD-L1 was detected in the culture supernatants of PD-L1 positive tumor cell lines but not in that of PD-L1 negative tumor cell lines. This result, obtained in vitro, shows that tumor cells release sPD-L1, but the release mechanism remains unclear. sPD-L1 could be actively shed by tumor cells or could be released by dying tumor cells. Although little data is currently available, we can hypothesize that sPD-L1 is actively shed by tumor cells as it was found that patients with decreasing sPD-L1 levels after nivolumab initiation presented with better outcomes [25]. The protein sequence of sPD-L1 has also been studied [18] by performing affinity chromatography using cell culture supernatants of PD-L1 positive tumor cells. The eluted fractions were tested with ELISA and the fractions with the highest sPD-L1 concentrations were pooled and subjected to protein electrophoresis and immunoblotted revealing a 45kDa band. Protein sequencing of this band revealed that it corresponded to the N-terminus part of PD-L1 and contained the Ig-V ligand-binding domain required for interaction with PD-1 on T-cells and delivering immunoinhibitory signals. Other studies [26] have shown that human peripheral blood mononuclear cells (PBMC), challenged with the mitogen PHA, released sPD-L1, suggesting that immune cell activation might be needed in order to produce sPD-L1. To further determine which cells secreted sPD-L1, PBMC were separated into plastic and non-plastic adherent cell. Plastic adherent cells (monocytes, macrophages, and DCs) released sPD-L1, but non-plastic adherent ones (T cells) did not. Moreover, activated CD3+ T-cells expressed mPD-L1 but did not release sPD-L1. These in vitro results strongly suggest that, among immune cells, sPD-L1 is secreted by monocytes, macrophages, and DCs, but not by T lymphocytes. Functional analyses showed that sPD-L1 increased the apoptotic activity of activated CD4 T cells and, to a lesser degree, of CD8 T cells. This suggests that sPD-L1 has biological activity and can deliver immunosuppressive signals to activated T cells [18]. Furthermore, as sPD-L1 has similar structural points to mPD-L1, we can hypothesize that it could bind with anti-PD-L1 drugs and, thus, exert a competing effect, explaining why patients with high levels of sPD-L1 have worse outcomes. Data to support this hypothesis has been reported by Gong et al. [27]. They found that patients who were resistant to ICIs had sPD-L1 variants that were secreted. These variants lacked the transmembrane domain usually present and cause ICI inefficacy by binding to the monoclonal antibodies and inhibiting their usual activity. These results are of the utmost interest and further studies are needed to confirm and validate them.

## 3. Other Soluble Proteins

### 3.1. Granzyme B

Granzyme B is a serine protease secreted by NK cells and cytotoxic CD8+ T cells, with a key role for immune-induced apoptosis. Granzyme proteins are delivered to the target cells via cytotoxic granules and are responsible for caspase-dependent apoptosis [28,29]. Several studies [30,31] have evaluated granzyme B activity through dedicated PET imaging in pre-clinical models showing that granzyme B activity can be used to predict response to ICIs and that tumors classified as high-signal with regards to granzyme B uptake presented with good response to ICIs.

Costantini et al. [25] found that soluble granzyme B (sGranzyme B) concentrations at nivolumab initiation in NSCLC patients were higher in patients who were responders than in non-responders (18.44 pg/mL vs. 11.88 pg/mL, *p* = 0.039). Significant results were also found when analyzing the variation of sGranzyme B during nivolumab treatment: patients with increasing concentrations of sGranzyme B between nivolumab initiation and the first tumor evaluation had worse outcomes with lower rates of ORR, shorter PFS (2.0 months vs. 8.8 months, *p* = 0.019), and OS (4.5 months vs. NR, *p* = 0.043) than patients with stable or decreasing concentrations. At nivolumab initiation, patients with high sGranzymeB concentrations had a non-reached median OS and a median PFS of 8.8 months, versus 4.5 months (*p* = 0.096) and 1.8 months (*p* = 0.018) respectively, for patients with low sGranzymeB concentrations. We hypothesize that high levels of pre-treatment sGranzyme B reflect an activated and efficient CD8+ cytotoxic immune response. The increase in sGranzyme B could be the reflection of an increasing yet ineffective T-cell response leading to T-cell exhaustion.

### 3.2. PD-L2, IL-2, IFN-Gamma

There is little data available on other soluble immune-related biomarkers, such as sPD-L2, interleukine (IL), or interferon-gamma (IFN-γ). We were not able to find any impact of the concentrations or dynamic evolution of these soluble proteins during nivolumab treatment on the efficacy of ICIs [25]. However, we showed that sPD-L2, sIL-2, and sIFN-γ concentrations at the early stages of nivolumab treatment were associated with the occurrence of an immune grade 3–4 toxicity with nivolumab. Another study [32] included 34 patients with advanced NSCLC who had failed at least one prior chemotherapy regimen and subsequently received nivolumab monotherapy (3 mg/kg every two weeks). Blood samples were collected at baseline, at week four, eight, twelve, and at the time of progression. Serum proteins were quantified by Milliplex MAP assay using human cytokine/chemokine panel 1, human angiogenesis/ growth factor panel 1, and a multi-species TGF-β panel (Millipore, Billerica, MA, USA). At week four, three proteins had different levels of expression between patients with irAEs and patients without irAEs: G-CSF, leptin, and RANTES. Multivariate analysis revealed that only the levels of RANTES were associated with irAEs. It was also shown that RANTES levels decreased after corticosteroid initiation. RANTES, also known as CCL5 (Chemokine ligand 5), plays an active role in recruiting leukocytes into inflammatory sites. It can also induce the proliferation and activation of natural killer (NK) cells using cytokines such as IL-2 or IFN-γ secreted by T-cells. Finally, IL-8 has been studied in melanoma and advanced NCLC patients [33]. Nineteen patients with NSCLC treated with nivolumab or pembrolizumab were included and it was found that responders had significantly decreasing levels of IL-8 between baseline and the first tumor evaluation while non-responders had significantly increasing levels of IL-8. Furthermore, an early decrease in IL-8 levels was associated with longer OS (not reached vs. 8.0 months 95% CI: 0–19.7, *p* = 0.015) compared to patients with early increasing levels of IL-8.

These results are mostly exploratory. However, the high levels of RANTES measured at week four in the study by Oyanagi et al. found to be associated with irAEs and the elevated concentrations of sIFN-γ found to be associated with irAEs by Costantini et al. are coherent results. In fact, an excessive T-cell reaction via a RANTES-mediated mechanism could lead to the excessive secretion of cytokines such as IL-2 and IFN-γ. The relatively high incidence of high-grade irAEs with ICIs (around 10–15%), sometimes associated with severe morbi-mortality or the interruption of ICI treatment underlines the necessity for the early detection of patients at risk for such complications.

## 4. Circulating Tumor DNA (ctDNA)

Circulating cell-free DNAs (cfcDNA) are short DNA fragments that are derived from dying cells. These fragments are present in healthy subjects where they are mostly derived from hematopoietic cells as well as in patients with cancer where a fraction can be derived from cancer cells [34]. In this case, the DNA fragments are called circulating tumor DNA (ctDNA). ctDNA can be explored by different ways: a quantitative approach in which ctDNA concentration is measured; a targeted sequencing approach (mostly by digital droplet PCR, ddPCR), to determine specific mutation profile as epidermal growth factor receptor (EGFR) mutations for example, or large ctDNA sequencing (next-generation sequencing, whole-exome sequencing) to have an extended view on mutational tumor profile and evaluate the mutational burden, also defined as blood tumor mutational burden (bTMB).

### 4.1. ctDNA Quantitative Approach and ICIs in NSCLC

A study by Cabel et al. [35] investigated whether ctDNA changes were correlated with outcomes in patients treated with anti-PD-1 therapy. This small study included 15 patients (10 treated for metastatic NSCLC, three treated for metastatic uveal melanoma and two treated for metastatic micro-satellite instable colorectal cancer) all receiving either nivolumab (3 mg/kg every 2 weeks) or pembrolizumab (2 mg/kg every three weeks). Plasma samples were prospectively collected at week 0 and week 8. In the case of a detectable mutation available in the patients’ record, it was used as the target to monitor plasma ctDNA using ddPCR or Bi-PAP depending on the mutation type. If no mutation was known, cfcDNA was submitted to targeted NGS. At baseline, ctDNA was detected in 10 out of the 15 patients (67%) and no association was found between baseline ctDNA levels and clinical characteristics. When analyzing ctDNA levels and tumor size change between week 0 and week 8, a statistically significant association was found (Spearman correlation r = 0.86, *p* = 0.002). Furthermore, patients with undetectable ctDNA at week 8 had significantly longer PFS than patients with detectable ctDNA (median PFS 11 vs. 2 months HR = 10.2, 95% CI: 2.5–41, *p* = 0.001) and OS (HR = 15, 95% CI: 2.5–95, *p* = 0.004). At baseline, patients with undetectable ctDNA levels had significantly longer OS durations (HR = 6.8, 95% CI: 1.1–41.0, *p* = 0.03) but no difference was found in terms of PFS.

These results were confirmed in another study where 49 patients with metastatic NSCLC treated with ICIs were included [36]. Plasma was drawn at baseline and then regularly throughout the follow-up period. Somatic mutations within cfcDNA were identified and quantified using a deep sequencing method and ctDNA was quantified using the allelic fraction of mutant tumor-derived DNA within total cfcDNA. If two mutants were detected, the one with the highest allelic fraction was used. Included in the study were 28 patients that had somatic mutations identified in their baseline plasma. ctDNA response was defined as a drop in ctDNA level to <50% of baseline confirmed by a second successive measurement. A strong correlation was found between ctDNA response and best radiographic response (Cohen’s kappa statistic [k = 0.753; 95% confidence interval (CI): 0.501–1.000, *p* < 0.001]). Out of the 24 patients achieving PR, 10 had a ctDNA response. The magnitude of the decrease in ctDNA levels was also greater in responders compared to non-responders (*p* = 0.002). Of interest, ctDNA response seemed to be achieved earlier than radiological response (24.5 days since the start of treatment vs. 72.5 days for radiological assessment). Finally, ctDNA responders had a longer duration of immunotherapy treatment (205.5 vs. 69 days, *p* < 0.001) and presented with a significantly lower risk of disease progression (HR = 0.29, 95% CI: 0.09–0.89, *p* = 0.03) and a lower, but not significantly, risk of death (HR = 0.22, 95% CI: 0.05–1.02, *p* = 0.053).

Finally, two studies have focused on patients with advanced NSCLC treated with nivolumab. The first study [37] calculated tumor volume (TV) by summing the diameter of five target lesions. Tumor tissue was obtained for all 14 patients included and plasma was drawn at baseline and at 1, 2, 4, 6, and 8 weeks after initiation of nivolumab treatment. The study found a significant correlation between TV and ctDNA levels (*p* = 0.02) and showed that ctDNA levels in responders usually decreased during follow-up whereas ctDNA in non-responders remained high after treatment initiation. The final study [38] included 20 patients with plasma drawn at initial diagnosis, at nivolumab initiation, and at first tumor evaluation. At baseline, ctDNA concentrations were not different between patients with objective response (OR) and patients without OR and were not correlated with baseline tumor burden. At first tumor evaluation, patients with OR had lower ctDNA concentrations than patients without OR (*p* = 0.032). Furthermore, responders all had decreasing ctDNA levels whereas 60% of non-responders had increasing ctDNA levels (*p* = 0.025). Finally, a ctDNA cut-off was determined at first tumor evaluation (0.006 ng/µL) with significantly longer PFS (*p* = 0.003) and OS (*p* = 0.044) in patients with lower ctDNA levels.

Taken together, these results support the fact that ctDNA could be a useful biomarker to predict response to ICIs in patients with advanced NSCLC. However, larger dedicated prospective trials are urgently needed, as so far data has only been collected from very small cohorts of patients, mostly in a retrospective way. Some technical limitations remain as ctDNA is not detected in all patients with advanced NSCLC and there is a need for standardization of the different ctDNA detection techniques. Finally, there seems to be little data [39] on the behavior of ctDNA in cases of particular progression patterns such as pseudo-progression or hyper-progressive disease.

### 4.2. Tumor Mutational Burden (TMB)

Tumor mutational burden can be defined as the number of somatic missense mutations present in a tumor sample. The optimal tool for measuring TMB is whole-exome sequencing (WES). As this is expensive and not widely available, NGS using cancer-gene panels (CGP) has also been used. Several CGP currently exist, such as Memorial Sloan Kettering Cancer Center’s Integrated Mutation Profiling of Actionable Cancer Targets (MSK-IMPACT), Foundation One CDx (FICDx), Guardant 360, PlasmaSELECT 64, and Foundation ACT.

TMB has revealed itself as a potential biomarker in melanoma, urothelial carcinoma, and lung cancer [40,41,42,43]. TMB was notably analyzed in an exploratory analysis of the Checkmate-026 study [41] which compared nivolumab to platinum-based immunotherapy in patients with PD-L1 positive advanced NSCLC. The study was negative on PFS (primary objective) among patients with a PD-L1 expression level of 5% or more (median PFS of 4.2 months with nivolumab versus 5.9 months with chemotherapy, *p* = 0.25). However, patients with high TMB (≥243 mutations as determined by WES) had improved PFS and ORR when they received nivolumab, compared to patients treated with chemotherapy. In Checkmate-227, NSCLC patients received either nivolumab-ipilimumab combination therapy or platinum-based chemotherapy in first-line treatment [42]. PFS was used as an endpoint in patients with high TMB (defined as ≥10 mutations/megabase determined by the FoundationOne CDx assay). In this population, immunotherapy combination was superior to chemotherapy with regards to PFS: HR = 0.58 (97.5% CI, 0.41–0.81), *p* < 0.001. However, analyses on TMB was not pre-defined, patients were not stratified on TMB, and only 57.7% of patients had TMB results available, making definitive conclusions on these results difficult.

Although promising, the clinical use of tissue TMB (tTMB) remains currently challenging, as it requires large amounts of tissue in order to perform effective sequencing, and there is still no standardization in term of technics or thresholds.

Less invasive methods have been sought, such as measuring TMB on ctDNA (bloodTMB or bTMB). Wang et al. [44] evaluated bTMB in patients with NSCLC using an NGS cancer gene panel. The study contained four sections: panel design, virtual validation, technical validation, and clinical validation. The CGP used was called NCG-GP150 and was designed by selecting cancer-related genes. In order to create the CGP, WES data from 9205 samples from The Cancer Genome Atlas (TCGA) were used and genes were randomly extracted to generate panels for TMB extraction. The more genes included, the higher the correlation between the CGP and the WES-based TMB. When 150 genes were used (NCC-GP150), a plateau was reached when evaluating the correlation between the CGP and the WES-based TMB. A validation cohort was used on 48 patients with advanced NSCLC and treated with anti-PD-1 ICI, showing a good correlation between NCC-GP150 and WES results, and a bTMB cut-off of six or higher for an optimal Youden index of 0.59 (0.88 sensitivity and 0.71 specificity). Finally, to evaluate the clinical significance of NCC-GP150, an independent cohort of 50 patients with advanced NSCLC and treated with anti-PD-1 or PD-L1 agents was analyzed. It was found that patients with high bTMB (>6) had longer PFS when compared to patients with low bTMB: median not reached vs. 2.9 months (HR = 0.39, 95% CI: 0.18–0.84, *p* = 0.01).

Another study [45] reported bTMB results using more than 1000 prospectively collected plasma samples from patients of the POPLAR [46] and OAK [4] studies. POPLAR was a phase II study that compared atezolizumab and docetaxel in the second-line setting for patients with previously treated advanced NSCLC. The OAK study was a randomized phase III trial with a similar setting. Both studies showed the superiority of atezolizumab versus docetaxel, in terms of OS regardless of PD-L1 expression or histology. tTMB and bTMB were evaluated by FoundationOne CDx NGS assay. Exploratory analyses from a subset of POPLAR and OAK samples showed a positive correlation between bTMB and tTMB (Spearman rank correlation = 0.64; 95% confidence interval (CI): 0.56–0.71). Patients with higher bTMB had improved PFS and OS benefit. For example, in the POPLAR study (n = 273), at the bTMB cut-off of 16 mutation/megabase, the median PFS in patients with bTMB >16 was 4.2 months in the atezolizumab arm and 2.9 months in the docetaxel arm with a HR of 0.57 (95% CI: 0.33–0.99). Similar results were found when using data from the OAK study.

### 4.3. Current Limitations for ctDNA and bTMB Use as Biomarkers for ICIs in NSCLC

Using ctDNA or bTMB to predict and track early response to ICIs is a promising approach. The studies described above and summarized in Table 2 have shown that the quantitative approach to ctDNA can help to predict responses to ICIs as early as the baseline and, maybe even more importantly, the early variation of ctDNA precisely predicts the response to immunotherapy. bTMB has also brought promising results, showing good correlation with tTMB and helping to predict response to ICIs in patients with high bTMB. However, these methods still present several limitations. First, ctDNA needs to be detected in the patients’ plasma. Furthermore, there are currently several panels used to determine bTMB, all using different gene targets, sequences, and thresholds leading to a lack of harmonization of research results. This is also an expensive technique and most data available to date is retrospective. Efforts are needed to improve the ctDNA detection rate, harmonize the different assays used, and to build clinical trials that will prospectively evaluate ctDNA and bTMB as a biomarker in lung cancer patients treated with ICIs.

## 5. New Plasma Biomarkers: Blood Microbiome and Plasma Marker of the Intestinal Barrier

It has been shown that gut microbiome influences the efficacy of PD-1-based immunotherapy in epithelial tumors [47]. As with the previous biomarkers reviewed, the blood microbiome is easily accessible and manageable as compared to fecal microbiome measurement [48,49,50]. Moreover, early use of antibiotics (EUA) has been proven to be associated with worse outcomes with ICIs, probably due to alteration in the intestinal barrier, modification of fecal microbiota, bacterial translocation, and modification of the anti-tumor immune response.

A recent pilot study described the plasma evaluation of the intestinal barrier and blood microbiome in a cohort of NSCLC patients treated with nivolumab. Ouaknine et al [51] included 72 consecutive patients with advanced NSCLC treated with nivolumab in a second-line setting or more. EUA was defined as oral or intravenous anti-biotherapy given two months before until one month after the beginning of nivolumab. Plasma citrulline, secreted by enterocytes and proven to be a biomarker of the intestinal barrier [52] was prospectively evaluated at months (M) M0, M1, M2, M4, and M6. The blood microbiome was assessed at baseline. It was found that patients with EUA had a shorter median OS (5.1 months vs. 13.4 months, *p* = 0.03) compared to patients without EUA. High baseline citrulline rates (≥20 μM) were associated with clinical benefit (*p* = 0.002), longer PFS (*p* < 0.0001), and longer OS (*p* < 0.0001). Patients with EUA had lower citrulline concentrations. Finally, different blood microbiome compositions were determined according to the tumor response and clinical benefit with nivolumab. For example, the presence of Gemmatimonadaceae DNA was associated with both low response rates (14% vs. 50% for other patients, *p* = 0.09) and high rates of progression at M2 (86% vs. 29% for other patients, *p* = 0.006). In multivariate analysis, low citrulline concentrations (HR = 3.8, 95%CI: 1.4–99.9, *p* = 0.008) was associated with worse PFS, and the presence of Gemmatimonadaceae DNA (HR = 16.4, 95% CI: 3.9–68.5, *p* < 0.001) and EUA (HR = 2.2, 95% CI: 1.1–4.8, *p* = 0.038) were associated with worse OS. Finally, a response-associated blood microbiome profile was more frequent in patients who did not receive EUA.

These preliminary results need to be confirmed in a prospective way, but open exciting new perspectives in ICI-related plasma biomarkers.

## 6. Future Directions and Conclusions

**Figure 1:**
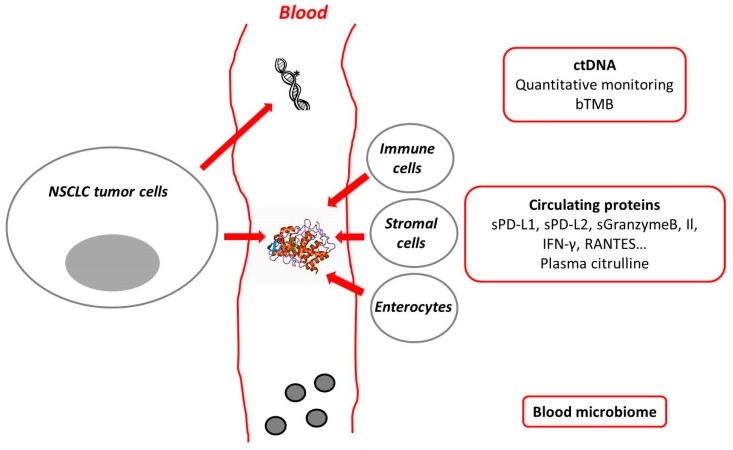
Plasma biomarkers and immune checkpoint inhibitors in non-small cell lung cancer.

Throughout this review, we have covered potential new plasma biomarkers such as soluble proteins, ctDNA, bTMB, citrulline, and blood microbiome (Figure 1). These biomarkers can help to better select patients who might respond to immunotherapy, they can help to determine an early response, prior to clinical or radiological evaluation and might even help in anticipating adverse events. Most of the available results are preliminary and cannot be implemented into daily clinical practice. The next step will be to develop prospective clinical trials testing different biomarkers, alone or in combination, in order to determine thresholds and different strategies to adopt. Challenges remain, as with PD-L1 IHC, a harmonization process will be needed for all biomarkers. As things stand, good quality biopsies are still required to choose the optimal treatment options. Available data suggest a comparable efficacy of tissue-based biomarkers for predicting ICI efficacy [53]. However, it is plausible that soluble biomarkers will soon be used as tools to help clinicians treating patients with advanced NSCLC receiving immunotherapy.

## Figures and Tables

**Table 1:** Comparison of the different studies testing soluble programmed death-ligand 1 (sPD-L1).

Study	Patients (n)	Time of sPD-L1 Measure	sPD-L1 Cut-Off	Treatment	Main Results
Zhang et al. [19]	Advanced NSCLC (109), healthy controls (65)	Diagnosis	0.636 ng/mL	NS	Higher sPD-L1 levels in patients than controls Shorter OS in high sPD-L1 patients
Okuma et al. [20]	Advanced or postsurgical recurrent lung cancer (96)	Before initiation of CT or at least 3–4 weeks after last CT	7.32 ng/mL	CT	Shorter OS in high sPD-L1 patients
Zhao et al. [23]	Locally advanced or inoperable NSCLC (126)	Diagnosis, Week 2 and 4 of treatment	0.0965 ng/mL	TRT ± CT	Decrease of sPD-L1 during TRT Shorter OS in high sPD-L1 patients
Okuma et al. [24]	Advanced or recurrent NSCLC (39)	Baseline	3.357 ng/mL	nivolumab	Shorter OS TTF in high sPD-L1 patients Higher rates of CR, PR, and SD in low sPD-L1 patients
Costantini et al. [25]	Advanced NSCLC (43)	At initial diagnosis, at nivolumab initiation, at first tumor evaluation	0.0337 ng/mL	nivolumab	sPD-L1 at first tumor evaluation higher in non-responders Patients with clinical benefit had lower sPD-L1 levels at first tumor evaluation Patients with increasing sPD-L1 levels had worse ORR, lower rates of clinical benefit, shorter PFS and OS

NSCLC: Non-small cell lung cancer. CT: Chemotherapy. TRT: Thoracic radiotherapy. NS: Not specified.

**Table 2:** Comparison of the different studies testing circulating tumor DNA (ctDNA) and blood tumor mutational burden (bTMB).

Study	Patients (n)	Variable Measured	Cut-Off Used for bTMB	Treatment	Positive Findings
Cabel et al. [34]	NSCLC, uveal melanoma, MSI colorectal cancer (10)	ctDNA concentrations	NA	nivolumab, pembrolizuamb	PFS, OS
Goldberg et al. [35]	Metastatic NSCLC (28)	ctDNA concentrations	NA	Anti PD-1, anti-PD-L1 alone or in combination	Time on treatment, PFS, OS
Iijima et al. [36]	Advanced NSCLC (14)	ctDNA concentrations	NA	nivolumab	Durable response
Giroux-Leprieur et al. [37]	Advanced NSCLC (15)	ctDNA concentrations by NGS	NA	nivolumab	PFS, clinical benefit
Wang et al. [43]	Advanced NSCLC (50)	NCC-GP150 (150 genes)	bTMB ≥ 6	Anti-PD-1 Anti-PD-L1	PFS ORR
Gandara et al. [44]	Advanced NSCLC (273)	NGS	bTMB ≥ 16	Atezolizumab (anti-PD-L1)	PFS

PFS: Progression-free survival. NSCLC: Non-small cell lung cancer. ORR: Objective response rate. bTMB: Blood tumor mutational burden. NA: Not applicable.
